# LRRC75A antisense lncRNA1 knockout attenuates inflammatory responses of bovine mammary epithelial cells

**DOI:** 10.7150/ijbs.38214

**Published:** 2020-01-01

**Authors:** Xixi Wang, Hao Wang, Ruiqi Zhang, Dan Li, Ming-Qing Gao

**Affiliations:** College of Veterinary Medicine, Northwest A&F University, Yangling 712100, China.

**Keywords:** bovine mammary epithelial cell, lncRNA, LRRC75A-AS1, LRRC75A, bovine mastitis

## Abstract

Long noncoding RNAs (lncRNAs) play multiple key roles during inflammatory processes. In this study, a novel lncRNA identified by the high-throughput sequencing analysis was found significantly down-regulated in *Escherichia coli*-introduced cell model of bovine mastitis. Given that this lncRNA consists of the antisense of leucine-rich repeat-containing protein 75A (LRRC75A), it was named LRRC75A antisense lncRNA1 (LRRC75A-AS1). The expression of LRRC75A-AS1 was down-regulated in bovine mammary epithelial cells and mammary tissues under inflammatory condition. Knockout (KO) of LRRC75A-AS1 by CRISPR-Cas9 system in bovine mammary alveolar cell-T (MAC-T) cell line could enhance expressions of tight junction (TJ) proteins Claudin-1, Occludin and ZO-1, reduce cell monolayer permeability, and inhibit *Staphylococcus aureus* adhesion and invasion. Meanwhile, it also down-regulated expressions of inflammatory factors and attenuated activation of NF-κB pathway. Similarly, knockdown of LRRC75A caused the changes as LRRC75A-AS1 KO did, while overexpression of LRRC75A enabled the opposite effects. TJ of epithelioid cells barriers the pathogenic microorganisms outside during inflammation, in which LRRC75A-AS1 can regulate the expression of TJ proteins through LRRC75A, affecting the development of inflammation.

## Introduction

Bovine mastitis is a common and frequently-occurring disease in dairy production systems worldwide and one of the top leading causes of milk production and milk quality decline. Once bovine mammary gland is infected with bacteria, it may affect production and quality of milk, and the most end up with huge economic losses. Bovine mastitis is mainly triggered by the infection of bacteria 1. Among all bacterial pathogenic microorganisms, *Escherichia coli* (*E. coli*) and *Staphylococcus aureus* (*S. aureus*) stand as the dominant pathogens 2, 3, but few effective measures against this disease was taken over years.

Long noncoding RNAs (LncRNAs) are transcripts with length of at least 200 nucleotides featuring limited protein-coding capability and poorly conserved sequence among mammalian species, and they account for about 98% of transcripts in human genome 4. LncRNA has been reported to be involved in various biological behaviors, such as the regulation of cell proliferation, apoptosis, autophagy and migration 5-8. Some lncRNAs are reported to involve in the modulation of maintain normal tissue structure and permeability 9-11. For instance, lncRNA metastasis associated lung adenocarcinoma transcript 1 is highly expressed in glioma cells and its knockdown increases the blood-tumor barrier permeability by up-regulating miR-140 10. LncRNA NEAT1 affects the expression of tight junction (TJ) proteins ZO-1, Occludin and Claudin-5 via regulating the miR-181d-5p/SOX5 pathway 11. Recent researches elucidate pivotal roles of non-coding RNAs during the inflammatory response 12, 13. LncRNAs could exert great influence in inflammation processes through JAK-STAT, MAPK and NF-κB pathways 14. LncRNA NKILA has been reported to inhibit activation of NF-κB pathway by binding IκB 15. LncRNA NEAT was found to participate in TLR4-mediated inflammatory processes induced by lipopolysaccharide via affecting activation of MAPK signaling pathways 16. Bidirectionally, lncRNAs are also regulated by inflammatory pathways 17, 18.

Epithelial TJ is an intercellular junctional complex which is composed of several proteins including Claudins, Occludins and ZOs. The dynamic change of this multi-functional complex is closely related with microbial adhesion, nutrition absorption, epithelial cell morphological structure, permeability and barrier function. Recent surveys have suggested that TJ proteins are associated with inflammatory bowel disease. It is clear that cytoskeletal regulation of barrier function is an important pathogenic process 19. When the disease occurs, the mucosal barrier function is abnormal, and then antigens shift to the lamina propria and activate immune cells, producing a large number of inflammatory cytokines and inflammatory medium. Various inflammatory factors and endotoxin can affect the expression of various TJ proteins; even destroy the junction complex finally increasing the permeability of intestinal mucosa 20, 21. It leads to an increase in cell gap permeability so that macromolecules such as bacteria and endotoxin can enter the systemic circulation through TJ.

Rare knowledge of lncRNA in bovine mastitis is known so far. In our study, lncRNA TCONS00021683 was screened out from a high-throughput sequencing analysis on *E. coli*-treated MAC-T cells. Multiple validations using quantitative real-time PCR (RT-qPCR) confirmed that TCONS00021683 was significantly down-regulated in the *E. coli*-treated cells and inflammatory mammary tissues. Since TCONS000021683 is the antisense of leucine-rich repeat-containing protein 75A (LRRC75A), we named it as LRRC75A antisense lncRNA1 (LRRC75A-AS1). TJs of epithelial cells can form a barrier to prevent the attack of pathogenic microorganisms during inflammation, and lower expression level of LRRC75A-AS1 lead to an enhancement of TJ structure, conducive to resisting the adverse microenvironment for bovine mammary during mastitis, which may serve as a potential therapeutic method in bovine mastitis.

## Materials and Methods

### Cell culture, treatment and tissue collection

Immortalized bovine mammary epithelial cells (MAC-T) 22 and primary bovine mammary epithelial cells (BMECs) isolated in our previous research 23 were cultured in complete DMEM/F12 medium (Gibco BRL, Grand Island, NY, USA) mixed with 10% fetal bovine serum, 100 IU/ml penicillin, and 100 µg/ml streptomycin (Gibco BRL) at 37°C in a humid incubator with 5% CO_2_. Inflammatory responses of MAC-T were induced by lipopolysaccharide (LPS) (Sigma-Aldrich, St Louis, MO, USA) at a final concentration of 10 ng/μl for 3 h or heat-inactivated *E. coli* at a ratio of 1:1000 according to our previous publications 24, 25. Tissues from Holstein dairy cows were extracted in our recent research 23.

### RT-qPCR and reverse transcription PCR (RT-PCR)

Tissues samples were completely grinded and then transferred to 1.5 ml non-enzyme EP tube, and cells were regularly harvested into EP tube. Total RNA was extracted from tissues and cells respectively using TriZol solution (TransGene, Beijing, China) in accordance with the manufacturer's instructions. RNA was reverse-transcribed into cDNA with the TransScript II First-Strand cDNA Synthesis Super Mix (TransGene). The RT-qPCR procedure was performed on ABI Step One Software System (ABI, Foster City, CA, USA) using SYBR Premix ExTaq II kits (Takara, Tokyo, Japan). For RT-PCR, PCR product was separated by 1% agarose gel. All PCR primers were designed by Primer Premier 5 (PREMIER Biosoft international, Palo Alto, CA, USA) and synthesized by TsingKe Biological Technology (Xian, Shaanxi, China). The primers are listed in Table S1. GAPDH or U6 were used as internal control.

### RNA protection assay

RNA extracted from cells was incubated at 37°C for 1 h, and then treated with RNase A (TransGene) for 30 min at 37°C. Single-stranded RNA was digested by RNase A, and remaining double-stranded RNA was reverse transcripted into cDNA and finally analyzed by RT-qPCR. To explore whether the overlapping and non-overlapping zone between LRRC75A and LRRC75A-AS1 were digested by RNase A, LRRC75A (1194 bp ~ 1339 bp) and LRRC75A (344 bp ~ 500 bp) were detected by RT-qPCR. The primers are listed in Table S1.

### Rapid-amplification of cDNA ends (3'-RACE)

The 3' end sequence of LRRC75A-AS1 was amplified by 3'-RACE method using a SMARTer™ RACE cDNA Amplification Kit (Takara) according to the manufacturer's instruction. For the first strand cDNA synthesis, the mRNA 3' end polyA tail was used as a primer binding site, and Oligo dT with a universal linker primer attached to the SMART oligonucleotide sequence as a locking primer. Then a gene specific primer was used as the upstream primer, and a universal primer containing a partial linker sequence as a downstream primer. The first strand cDNA was used as a template in PCR.

### Knockout of LRRC75A-AS1

PX459M (Miaoling, Wuhan, Hubei, China) and EZ-GuideXH (Miaoling) were used to construct the knockout (KO) vector backbone. Two small guide RNAs (sgRNAs) targeting two genome sites of LRRC75A-AS1 selected at http://chopchop.cbu.uib.no/, were respectively ligated into the two plasmids 26, 27. All the sgRNA sequences used were attached in Table S2. Through enzyme digestion and connection, KO vector was constructed and transfected into MAC-T cells. At 24 h after electrotransfection, puromycin (1.5 μg/ml) was added into medium to select out those drug-resistant cells containing KO plasmid within the next two days. For screening the final genome-edited cells, the survivals after puromycin treatment were diluted in 96-well plates at a density of one cell/well. When the single cell multiplied and shaped to a single island-like clone consisting of several hundreds of cells, each clone was digested and transferred to one well of 24-well plates. Finally, until cells grew to over 80% confluence, half of them were taken for PCR to identify the KO-positive cell clone, the remaining half continue to expand. The cell transferred with empty vector was regarded as control (KO-Control). KO of LRRC75A-AS1 was checked by genomic PCR using forward primer-GACGATAGTTTTCCCGACTGAC, GTCAGTCGGGAAAACTATCGTC and reverse primer-CCGTAGGTTCACCACTACACAA and PCR product size of the wildtype is 842 bp. The DNA bands with length of less than 800 bp in the gel were cut and sent to TsingKe Biological Technology (Xian, Shaanxi, China) for Sanger sequencing.

### Cell nucleus and cytoplasm fraction isolation

For protein and RNA extraction from cell nucleus and cytoplasm, PARIS™ Kit (Thermo Fisher, MA, USA) was used according to the manufacturer's instruction. First, collected cells were washed with PBS on ice. Then 500 µl ice-cold Cell Fractionation Buffer was used to resuspend cells. Standing on ice for 5 min later, the resuspended cells were centrifuged with 500×g at 4°C to separate the nuclear and cytoplasmic cell fractions. The supernatant was aspirated away from the nuclear pellet, and put in a fresh RNase-free tube for cytoplasmic protein detection. Then 500 μ1 ice-cold Cell Disruption Buffer was added to the nuclear pellet to be ready for nuclear protein detection. For cytoplasmic RNA isolation, 2×Lysis/Binding Solution and RNAqueous was added to cytoplasm. For nuclear RNA isolation, 2×Lysis/Binding Solution and RNAqueous was added after cell disruption buffer was added into the nuclear pellet. Finally both parts of RNA were purified based on the instructions.

### Western blotting

Cells were cultured and grown to around 80% confluence, then lysed with PRO-PREP Protein Extraction Solution (iNtRON Biotechnology, Inc. Gyeonggi-do, South Korea) or PARIS™ Kit. All protein concentrations were detected using a BCA Protein Assay Kit (Beyotime, Shanghai, China), and 30 μg of each sample was loaded. After resolved proteins were blotted onto PVDF transfer membranes and blocked with 10% non-fat milk in TBST for 2 h, the membranes were incubated with primary antibodies against ZO-1 (Bioss, Beijing, China), Occludin (Bioss), Claudin-1 (Bioss), p65 (Santa Cruz, Dallas, TX, USA), phosphorylated p65 (Bioss), GAPDH (Bioss) at 4°C overnight. After washed with PBS subsequently, the membranes were incubated with their corresponding secondary antibodies (Beyotime). In addition, 5% BSA (Beyotime) was used to block the membrane in phosphorylated p65 detection.

### Immunofluorescence

Cells were seeded into 24-well plates with density of 2x10^5^/well and cultured for 3 h. Paraformaldehyde was added to fix cells, and the fixed cells were treated with TritonX-100 (0.5%). Then cells were incubated with specific immunofluorescence antibodies overnight, after which incubated with the secondary antibody for 2 h in the dark. DAPI was used to stain nuclear. Labeled antibodies and nuclear were photographed by laser confocal microscope (Zeiss LSM800, Dresden, Germany).

### Knockdown and overexpression of LRRC75A

LRRC75A siRNA was designed and synthesized by Gene Pharma (Shanghai, China). The siRNA sequence interfering with LRRC75A gene (Si-LRRC75A) was 5'-CCGUGGACCUGUCAGGCAUTT-3' and the negative control sequence was 5'-UUCUCCGAACGUGUCACGUTT-3'. The full-length LRRC75A gene sequence downloaded from NCBI was cloned and verified by sequencing. The PCR product was digested with the BamH I and EocR I enzymes and ligated into CD513B-1 vector (System Biosciences, Mountain View, CA, USA), yielding the Over-LRRC75A plasmid. Then plasmids and siRNA were respectively transferred into cells by electroporation. Si-LRRC75A and Over-LRRC75A cell clones were verified by RT-qPCR.

### Luciferase Reporter Gene Assay

PGL4.10-NF-κB (Promega, Beijing, China) and pRL-TK-luc (Promega) at a ratio of 10:1 were transferred into cells. Twenty four hours later after transfection, cell lysates were used to determine luciferase activities of firefly and renilla by the dual luciferase reporter gene assay (Beyotime) according to the manufacturer's instructions. Firefly luciferase activity was normalized to renilla luciferase activity.

### Bacterial adhesion and invasion

Bacteria adhesion and invasion experiments were performed according to the method of Bianchi 26. For bacteria adhesion, cells with a density of 10^5^ cells/well were treated by 10^7^ FITC-labeled *S. aureus* in 24-well plate at 37°C for 2 h. After washing the cells with PBS, 300 μl 0.25% trypsin was added. Then 100 μl of digested cells were taken for measuring the fluorescence amount A_1_. The mixed solution containing 10^7^ FITC-labeled bacteria, 300 μl trypsin and 300 μl non-antibiotics DMEM medium was taken 100 μl to detect the amount of fluorescence (A_2_). The mixed solution containing 300 μl of trypsin and 300 μl of DMEM medium was taken 100 μl to measure fluorescence (A_0_) as blank control.

For bacteria invasion, after cells were co-incubated with FITC-labeled *S. aureus*, cephalosporin and gentamicin (100×) were added to kill the bacteria which did not invade into any cells. Then the cells were digested and lysed by 300 μl TritonX-100 for 10 min. Next steps of fluorescence detection were performed as that in bacteria adhesion essay. Fluorescence (A_0_) of 100 μl Triton X-100 was considered as blank control. Five repetitions were performed for all samples. Both cell adhesion and invasion rate were calculated by the formula: (A_1_-A_0_)/ (A_2_-A_0_) ×100%.

### Cell monolayer permeability assay

KO-Control and KO-LRRC75A-AS1 cells were seeded onto the transwell chambers fitted in a 6-well plate (0.3 μm pore size, 24 mm diameter; Corning Costar Corporation, Cambridge, MA, USA) with a density of 10^5^ cells per well. Until the cells formed a monolayer, culture medium was replaced with 1.5 ml medium containing low sugar DMEM without phenol red. Then 0.5 ml permeabilization test medium containing 300 ng horse radish peroxidase (HRP) was added into a transwell chamber. After incubation at 37°C for 0.5 h, 50 μl medium was taken from each well of 6-well plate and transferred to a 96-well plate. Then 50 μl tetramethylbenzidine (50 μg/ml) and 50 μl H_2_O_2_ (0.003%) were added in tandem to the wells, the process of which was neutralized by adding 50 μl 2 mol/L H_2_SO_4_. The absorbance (A) was measured at 450 nm by microplate reader. With A value and absorbance-HRP standard curve (A=0.176C+0.0716; R^2^=0.9999), the concentration of HRP passing through the cell monolayer formed by MAC-T cells or KO-LRRC75A-AS1 cells were calculated by the formula: HRP permeation rate (%) = (C_6-well plate_ × V_6-well plate_)/ (C_transwell_× V_transwell_), where C stands for concentration of HRP, and V stands for volume of the medium.

### Statistical analysis

All data with mean ± SD were obtained from at least three replicas of experiments. The statistical significance between two groups was measured with Student's t-test. P value<0.05 was considered statistically significant.

## Result

### LRRC75A-AS1 was down-regulated in bovine mammary epithelial tissues and cells under inflammatory conditions

**Figure 1:**
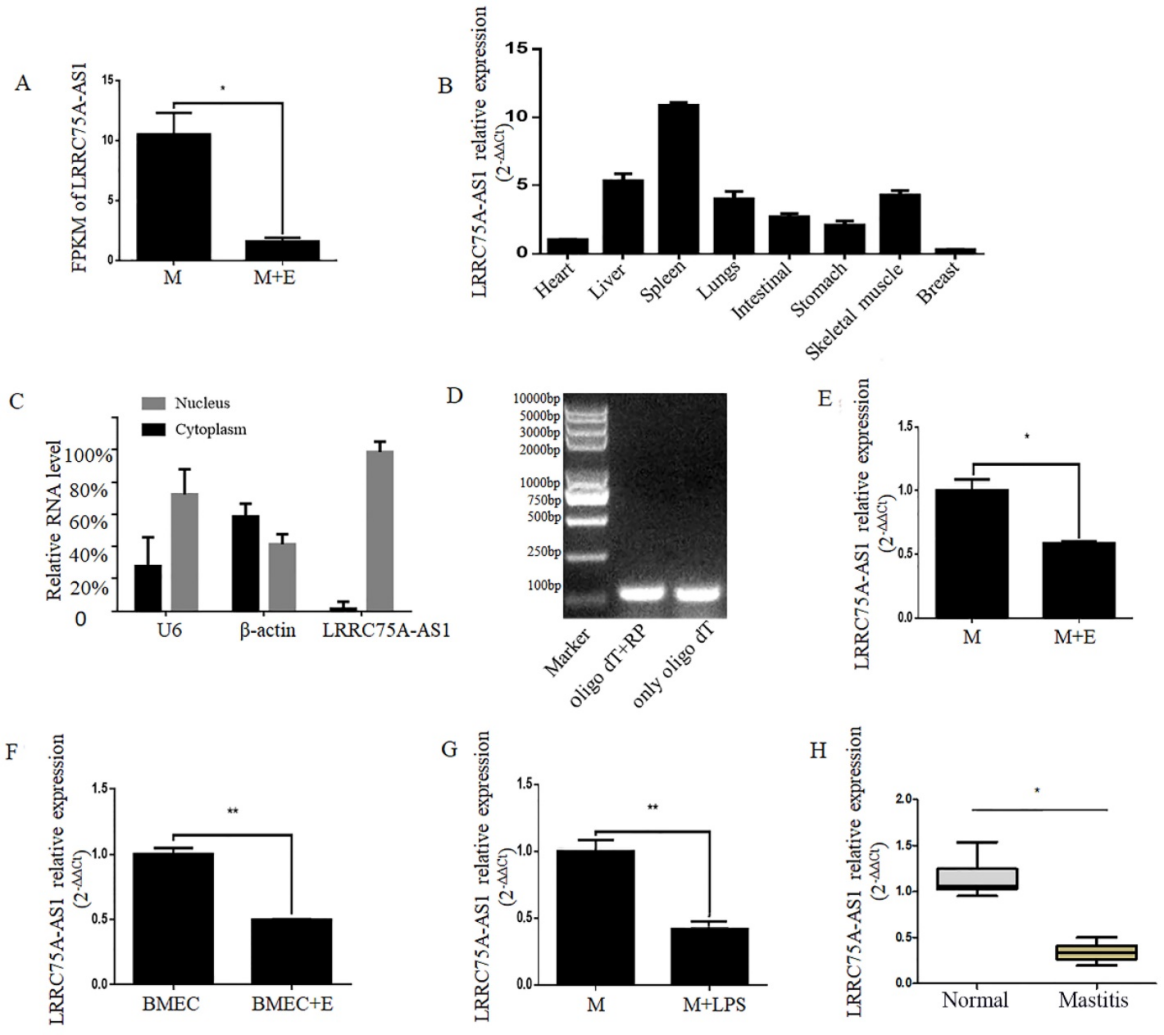
** Validation of LRRC75A-AS1 in cells and tissues by RT-qPCR. (A)** FPKM value of LRRC75A-AS1 in MAC-T (M) and MAC-T treated with heat-killed *E. coli* (M+E) was obtained from RNA sequence data. **(B)** Expression of LRRC75A-AS1 in eight different tissues was detected by RT-qPCR. **(C)** LRRC75A-AS1 was detected in nucleus and cytoplasm by RT-qPCR. **(D)** It was detected that if there is a polyA tail at 3'end of LRRC75A-AS1 by RT-PCR. Both Oligo dT and random primer (RP) were added in the reverse transcription reaction to reverse all transcripts, while Oligo dT only was used to reverse transcript RNA with polyA tail to cDNA for PCR, then the PCR products were separated respectively in lane 1 and lane 2. **(E)** LRRC75A-AS1 was detected in M and M+E by RT-qPCR. **(F)** LRRC75A-AS1 was detected in primary bovine mammary epithelium cells (BMECs). **(G)** LRRC75A-AS1 was detected by RT-qPCR after M was treated with LPS. **(H)** LRRC75A-AS1 was detected in normal bovine mammary tissues and mastitis tissues. **P<0.01, *P<0.05 vs control.

A novel lncRNA TCONS00021683 named as LRRC75A-AS1 was selected from a high-throughput sequencing analysis in which lncRNA expression profile was analyzed on *E. coli-*treated MAC-T cells 25, and it was found significantly down-regulated (Fig. 1A). Because of its higher fragments per kilobase of transcript per million fragments mapped (FPKM) and larger fold change among all the differentially expressed lncRNAs, LRRC75A-AS1 was selected for further study.

LRRC75A-AS1 is an antisense lncRNA located on chromosome 19. To explore the tissue specific expression of LRRC75A-AS1, eight tissues of cattle including heart, skeleton muscle, lung, liver, intestinal, stomach, spleen, and mammary gland of bovine were collected to detect the lncRNA by RT-qPCR. It was found that the highest expression was in the spleen and the lowest was in the breast among eight bovine tissues (Fig. 1B). In addition, most of LRRC75A-AS1 distributed in the nucleus (Fig. 1C) and was tailed with polyA (Fig. 1D), which was also confirmed by the result of 3'RACE (Additional file 1).

To validate the LRRC75A-AS1 expression from the high-throughput sequencing data, the lncRNA was further examined in mammary epithelial cells and mammary tissues under normal or inflammatory condition. The results of RT-qPCR showed LRRC75A-AS1 expression was down-regulated in *E. coli*-treated MAC-T cells (Fig. 1E),* E. coli*-treated primary mammary epithelial cells (Fig. 1F), LPS-treated MAC-T cells (Fig. 1G) and inflammatory mammary tissue (Fig. 1H) compared to their corresponding controls, all of which are consistent with the high-throughput sequencing data.

### KO of LRRC75A-AS1 changed cell morphologies

**Figure 2:**
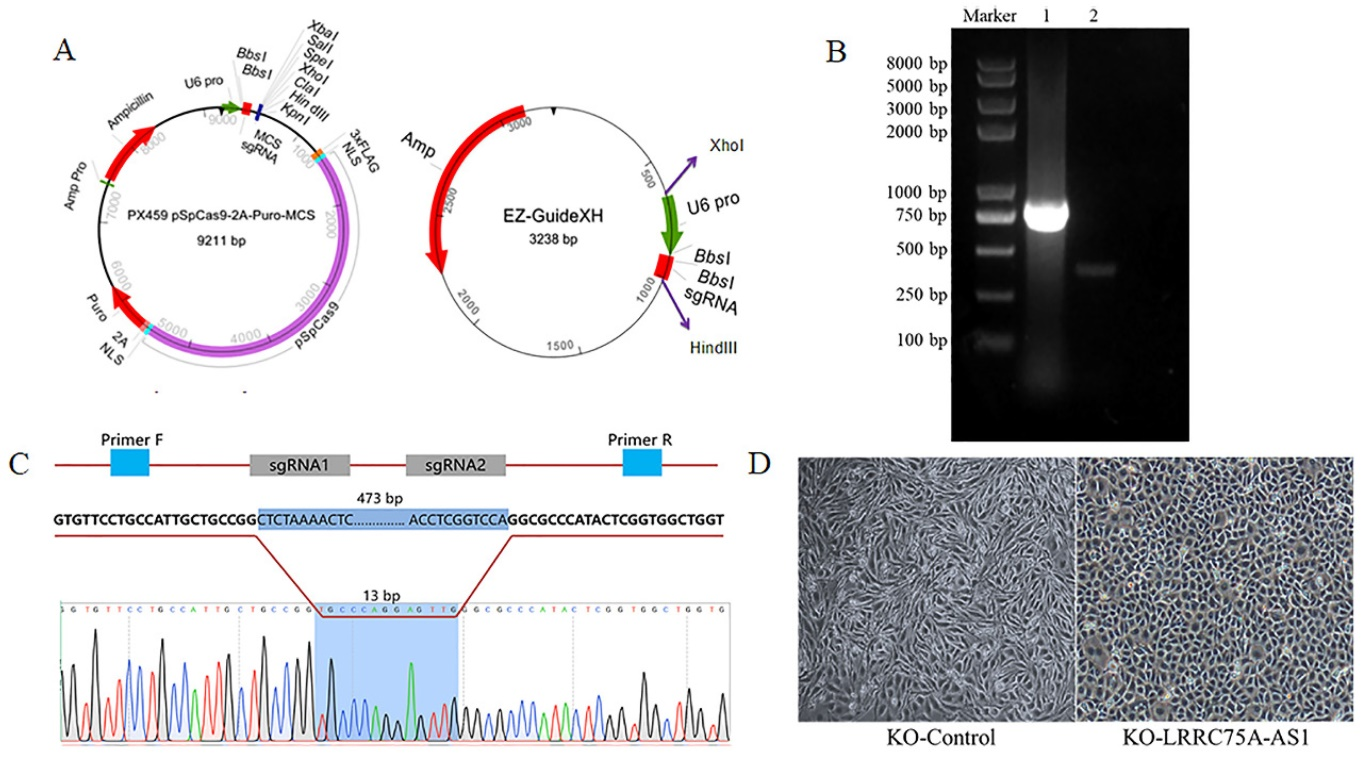
** LRRC75A-AS1 was successfully knocked out, verified by RT-PCR and Sanger sequencing. (A)** Plasmid maps of the PX459M and EZ-GuideXH were showed. Bbs I site in both plasmids was used to ligate sgRNA. Xho I and Hind III were used to ligate the digested smaller fragment of EZ-GuideXH containing U6 promoter and sgRNA to PX459M. **(B)** KO-LRRC75A-AS1 cell was verified by RT-PCR, which showed homozygous deletion of LRRC75A-AS1. Lane 1 showed the wildtype cell, and lane 2 showed KO cell. **(C)** A schematic graph explains genome editing strategy with two sgRNAs to knockout LRRC75A-AS1, and Sanger sequencing of the cell clone reported deletion of part of the LRRC75A-AS1 gene sequence. **(D)** The change of cell morphology was observed under phase contrast microscope.

To knockout LRRC75A-AS1, two optimized sgRNAs were designed and constructed into the two plasmids, then the fragment containing U6 and sgRNA in EZ-guidexXH was ligated into PX459M to construct a KO plasmid (Fig. 2A), and cells containing KO plasmid were screened out by puromycin. DNA electrophoresis of PCR product demonstrated that LRRC75A-AS1 gene in the clone was successfully knocked out 460 bp, compared to the wildtype (Fig. 2B). Sanger sequencing result indicated that there are extra 11-bp DNA deletions from the designed Cas9-sgRNA1-cutting site, 4-bp DNA deletions from the designed Cas9-sgRNA2-cutting site, and 13-bp DNA insertion linking two breaking ends (Fig. 2C, Additional file 1 and Additional file 2), which often happens when cell repairs double-stranded breaks caused by Cas9 28. After LRRC75A-AS1 KO, it was found that when the cells grows into a successive monolayer, intercellular space of LRRC75A-AS1^-/-^ cells was smaller than that of MAC-T cells, and LRRC75A-AS1^-/-^ cells were distributed more closely and orderly compared to control (Fig. 2D).

### KO of LRRC75A-AS1 enhanced TJ structure and decreased cell monolayer permeability

**Figure 3:**
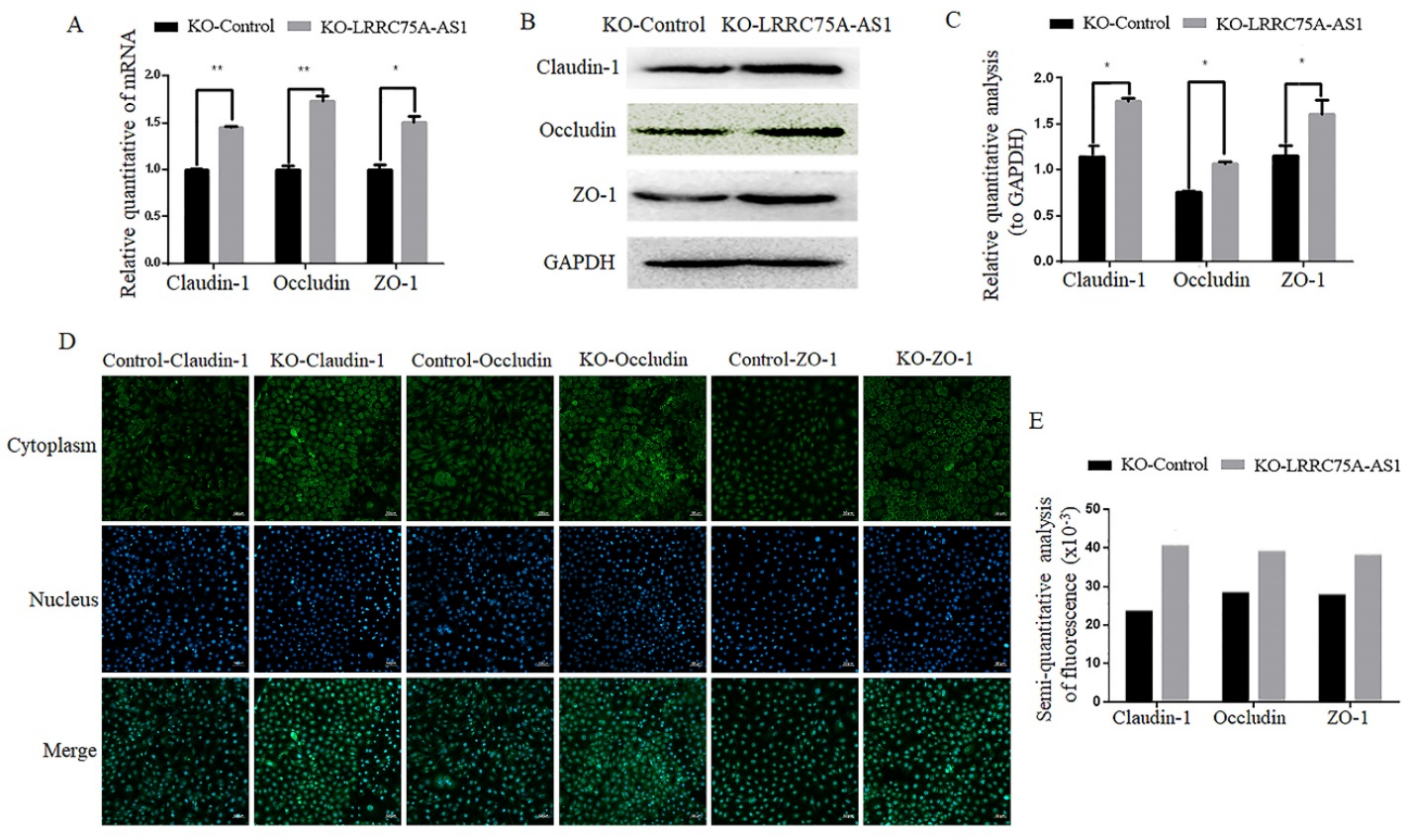
** KO of LRRC75A-AS1 enhanced TJ of MAC-T cells. (A)** Gene expressions of Claudin-1, Occludin and ZO-1 were analyzed by RT-qPCR. **(B)** Claudin-1, Occludin and ZO-1 expression in KO-Control and KO-LRRC75A-AS1 cells were analyzed by western blot. GAPDH was used as an internal control. **(C)** The relative quantitative analysis of western blot result was performed. *P<0.05, **P<0.01. **(D)** Immunofluorescence of Claudin-1, Occludin and ZO-1 was observed under laser confocal microscope. Nuclei were stained with DAPI. Scale bar: 50 µm. **(E)** The semiquantitative analysis of immunofluorescence was performed by using Image J.

Considering the morphology change and longer time for LRRC75A-AS1^-/-^ cells to be digested with trypsin relative to KO-Control cells observed (data not shown), we speculate LRRC75A-AS1 could affect the TJ of MAC-T cells. To validate the hypothesis, TJ proteins including Claudin-1, Occludin and ZO-1 at the mRNA and protein level were detected. As the results of RT-qPCR (Fig. 3A), western blot (Fig. 3B and 3C) and immunofluorescence (Fig. 3D and 3E) indicated, the levels of the three proteins were increased with different degree in LRRC75A-AS1^-/-^ cells compared to KO-Control cells. TJ proteins is closely linked with permeability of cell barrier 29. Cell monolayer permeability assay evidenced that the permeability rate value was lower (*p* < 0.01) in the KO of LRRC75A-AS1 group (0.25±0.003) than the KO-Control group (0.31±0.013) (Table 1). All these finding suggested the loss of LRRC75A-AS1 could affect the TJ structure to reduce cell barrier permeability, resisting the microorganisms from outside environment.

### KO of LRRC75A-AS1 weakened *S. aureus* adhesion and invasion

TJ structure plays an important role in the interaction between bacteria and epithelial cells 30. To explore if KO of LRRC75A-AS1 affected bacteria adhesion and invasion through the change of TJ structure, *S. aureus* adhesion and invasion experiments were performed. Results showed that the value of adhesion percentage of LRRC75A-AS1^-/-^ cells (5.68±0.31) was significantly lower (*p* < 0.01) than that of KO-Control (7.44±0.81), and invasion percentage value (2.14±0.11) was also significantly decreased (*p* < 0.01) compared to control (2.65±0.15) (Table 2), which implied that the LRRC75A-AS1^-/-^ cells were more resistant to bacterial adhesion and invasion.

### KO of LRRC75A-AS1 attenuated the *E. coli*-induced inflammatory responses

**Figure 4:**
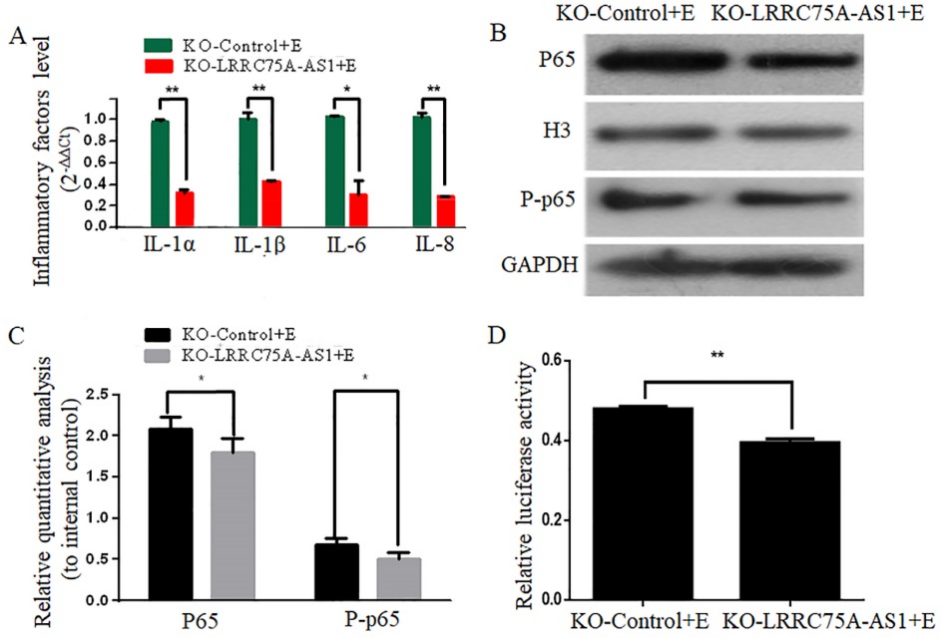
** KO of LRRC75A-AS1 attenuated the activation of inflammation. (A)** Inflammatory factors were detected by RT-qPCR. **(B)** Western blot on nuclear NF-κB subunit p65 and total phosphorylated p65 was performed to detect the activation of NF-κB pathway. **(C)** Relative quantitative analysis was performed. H3 was used as control in nucleus. GAPDH was regarded as control in cytoplasm. **(D)** The activation of NF-κB pathway was tested by dual luciferase reporter gene assay.

To investigate whether KO of LRRC75A-AS1 affected the *E. coli*-induced inflammatory response, expressions of inflammation-associated genes and the activation of NF-κB pathway were detected after LRRC75A-AS1^-/-^ cells were treated with heat-killed *E. coli*. We found that KO of LRRC75A-AS1 reduced the *E. coli*-introduced expression of inflammation-related genes including IL-1α, IL-1β, IL-6 and IL-8 (Fig. 4A).Western blot showed the nuclear NF-κB subunit p65 and total phosphorylated p65 were down-regulated in the LRRC75A-AS1^-/-^ cells compared with KO-Control (Fig. 4B and 4C), and the dual luciferase reporter gene assay showed weaker fluorescence signal of NF-κB pathway in LRRC75AAS1^-/-^ cells relative to that of KO-Control (Fig. 4D), all of which demonstrated that KO of LRRC75A-AS1 suppressed NF-κB pathway activation.

### LRRC75A-AS1 regulated LRRC75A mRNA expression

**Figure 5:**
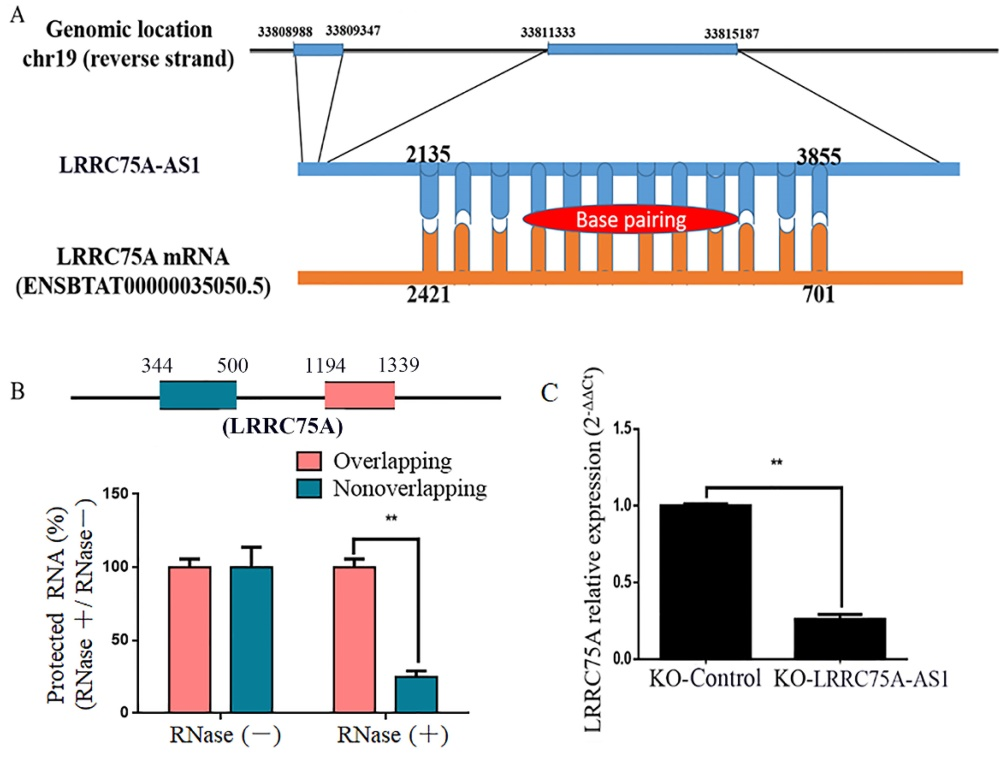
** The target of LRRC75A-AS1 (LRRC75A mRNA) was predicted and detected. (A)** There is 1720-bp base pairing region between LRRC75A-AS1 and LRRC75A mRNA found by RNAplex. **(B)** RNase protection assay was performed on RNA samples from MAC-T cells. The overlapping and non-overlapping regions of LRRC75A mRNA were detected by real-time qPCR. **(C)** LRRC75A mRNA in KO-LRRC75A-AS1 cells was detected by RT-qPCR.

By blasting the DNA sequence of LRRC75A-AS1 in bovine genome (http://genome.ucsc.edu/cgi-bin/hgBlat), it was found that LRRC75A is its neighbor gene. Furthermore, RNAplex software analysis (https://omictools.com/rnaplex-tool) showed that there was a 1721-bp complementary region between the sequence of LRRC75A mRNA and LRRC75A-AS1 (Fig. 5A). Antisense lncRNAs can up-regulate the expression of adjacent genes by increasing the stability through forming RNA-RNA dimers with its sense mRNAs 31, 32. RNA protection assay results showed that the overlapping fragment of LRRC75A and LRRC75A-AS1 still can be detected with a much higher level than that of non-overlapping fragment after RNase A digestion (Fig. 5B). Moreover, to investigate whether LRRC75A-AS1 affected its expression, LRRC75A was detected after LRRC75A-AS1 knockout. RT-qPCR result told that expression of LRRC75A in LRRC75A-AS1^-/-^ cell was reduced to a quarter of that in the KO-Control (Fig. 5C), suggesting LRRC75A-AS1 may protect LRRC75A from degradation by binding its CDS region.

### LRRC75A affected the TJ structure and cell monolayer permeability

**Figure 6:**
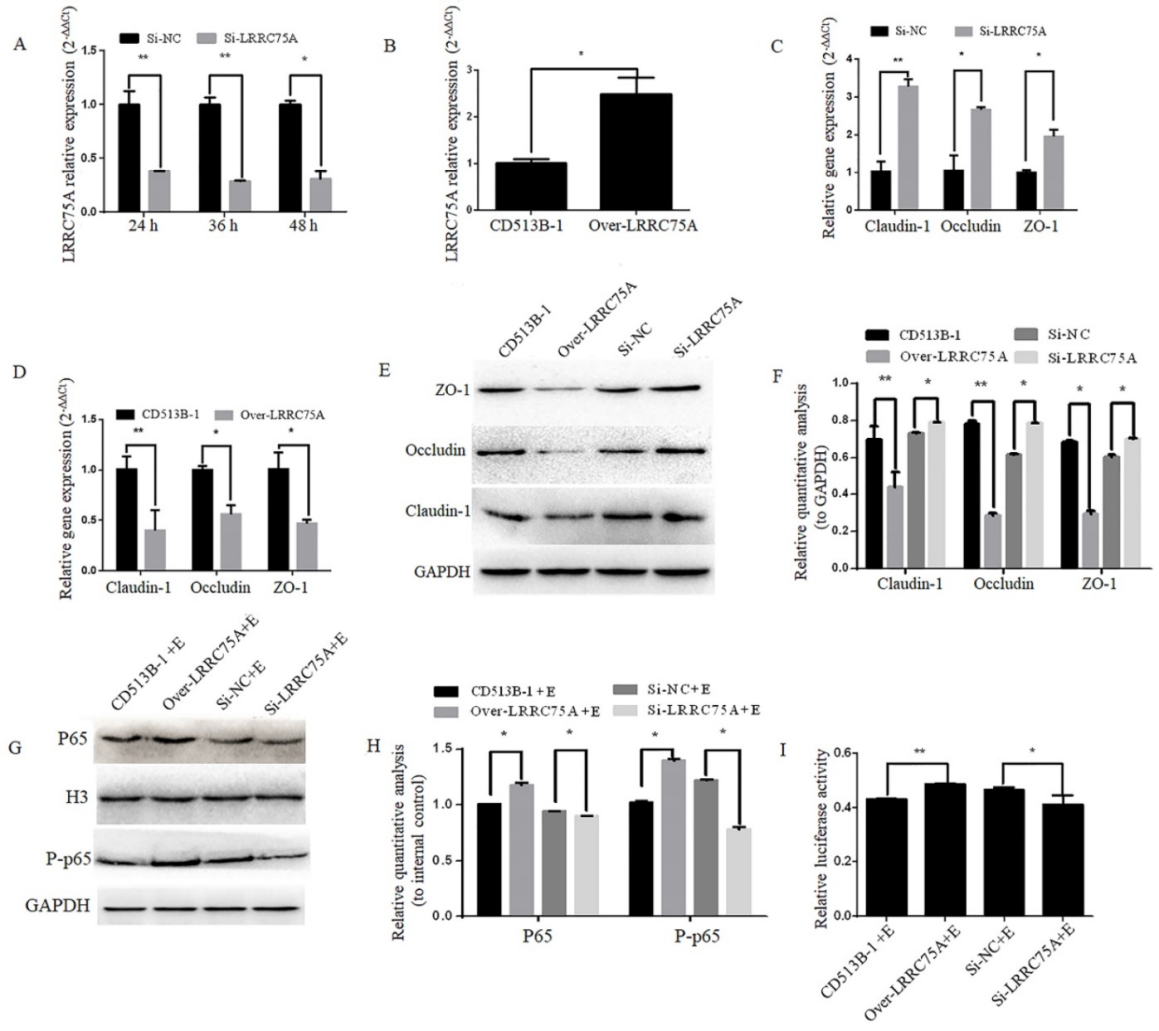
Lower expression of LRRC75A enhanced TJ and weakened the activation of inflammation. **(A)** LRRC75A was detected at indicated time point by RT-qPCR after siRNA was transfected into cells. **(B)** LRRC75A was overexpressed by CD513B-1 plasmid and detected by RT-qPCR. **(C) and (D)** The mRNA levels of Claudin-1, Occludin and ZO-1 were detected by RT-qPCR after siRNA and overexpression plasmid were respectively transfected into cells. **(E)** TJ proteins were detected by western blot after siRNA and overexpression plasmid were respectively transfected into cell. **(F)** Relative quantative analyze of TJ proteins in western blot was performed. **(G)** P65 in the nucleus and total P-p65 was detected by western blot. H3 was used as control in nucleus. GAPDH was regarded as control in cytoplasm. **(H)** Relative quantative analyze of p65 in the nucleus and P-p65 in western blot was performed. **(I)** Activation of NF-κB pathway was measured by dual luciferase reporter gene assay. (NC: Negative Control; Si: SiRNA knockdown; CD513B-1: Empty vector; Over: Overexpression.)

To figure out if LRRC75A, as the binding target of LRRC75A-AS1, could affect TJ structure, LRRC75A was knocked down and overexpressed as showed by RT-qPCR in Fig. 6A and Fig. 6B, after which TJ-related genes expression levels were detected by RT-qPCR and western blot. We found that LRRC75A knockdown up-regulated the mRNA level of Claudin-1, Occludin and ZO-1 (Fig. 6C), and the overexpression of LRRC75A produced the opposite result (Fig. 6D). Meanwhile, protein level of these genes was increased in LRRC75A knockdown cells, and decreased in LRRC75A overexpression cells (Fig. 6E and 6F). The cell monolayer permeability assay showed that the figure of permeability rate was lower (*p* < 0.01) in LRRC75A knockdown cells (0.22±0.003) than that of control (0.25±0.004), while in the LRRC75A-overexpressed cells group (0.31±0.004) was much greater (*p* < 0.01) than that of control (0.25±0.0002) (Table 3).

### LRRC75A affected the process of* S. aureus* adhesion and invasion

We next analyzed the role of LRRC75A in the process of *S. aureus* adhesion and invasion. The results demonstrated that adhesion percentage value in the LRRC75A knockdown cells (3.88±0.04) was lower (*p* < 0.01) than that of Si-NC group (4.07±0.03). Invasion percentage rate figure after LRRC75A knockdown (1.87±0.06) was also lower (*p* < 0.01) than that of control (2.37±0.06). In LRRC75A overexpression cells, the value of adhesion percentage (9.03±0.27) was higher (*p* < 0.01) than that of control (7.40±0.11), and the value of invasion percentage (2.91±0.06) was greater (*p* < 0.01) than that of control (2.49±0.04) (Table 4). These results supported that LRRC75A could affect the process of *S. aureus* adhesion and invasion to cells.

### LRRC75A was participated in the *E. coli*-induced activation of NF-κB pathway

To explore if LRRC75A was participated in NF-κB pathway, western blot was performed and it indicated that LRRC75A knockdown led to the reduced expression of nuclear p65 and total phosphorylated p65, while overexpression of LRRC75A promoted the activation of NF-κB pathway (Fig. 6G and 6H). Dual luciferase reporter experiment showed that the fluorescence signal of NF-κB pathway in LRRC75A knockdown cells is weaker than that of control, while the overexpression generated the opposite result (Fig. 6I). It suggested that LRRC75A may play a pivotal role during the *E. coli*-induced activation of NF-κB pathway.

## Discussion

LncRNAs have been documented to exert regulatory roles in a wide spectrum of diseases. Recent transcriptomics efforts have demonstrated that antisense lncRNA can rapidly and reversibly up-regulate the abundance of the sense mRNA in response to a variety of stresses 31. Here, we identified a conserved noncoding antisense transcript of LRRC75A (LRRC75A-AS1) as a bovine mastitis related lncRNA, which could influence the development of inflammation by regulating the expression of TJ proteins. LRRC75A-AS1 is a ~4-kb RNA transcribed from the opposite strand of the LRRC75A locus on chromosome 19. In order to understand its function in bovine mammary epithelial cells, LRRC75A-AS1 was knocked out by CRISPR-Cas9 system. Losses of the lncRNA directly give rise to some significant phenotypic changes. Tighter intercellular space of LRRC75A-AS1^-/-^ cells was observed under microscope. After LRRC75A-AS1 knockout, the cell monolayer permeability was much lower and adhesion and invasion of *S. aureus* to the cells were inhibited compared to control, while the activation of inflammatory pathways was also attenuated. To explain all these phenomena, we detected the TJ-associated proteins (Claudin-1, Occludin, ZO-1), and it was found that KO of lncRNA enhanced the expression of these proteins with different extent. The TJ, made up of two major transmembrane spanning structural proteins called Occludin and Claudin 33, 34, play an important role in the immune response during mastitis 35-37. Claudins are dynamic and adaptable system facing extracellular stimulus 38, and mainly contribute to the paracellular permeability of the TJ in epithelial and endothelial cells forming single cell layers 39, which are also participated in LPS-introduced mastitis 40. Occludin can protect epithelia monolayer from invading of pathogenic agents by shedding off the infected cell 41, 42. The interaction of Occludin and Claudin has been reported to be important for maintaining the barrier properties of a compromised epithelial monolayer during inflammation 42. ZO-1, as one of the skeleton proteins, has been reported to link the Occludin and Claudin to actin cytoskeleton 43. In summary, TJ protein plays an important part in the interaction between bacteria and cells. Therefore, it is tempting to hypothesize that the aberrant expression of Claudin-1, Occludin and ZO-1 in LRRC75A-AS1^-/-^ cell could enhance the TJ structure to reduce cell monolayer permeability and bacteria adhesion and invasion, thus restraining the bacteria particles from passing through TJ, which finally attenuated the inflammation-related pathways activation and the followed expression of many inflammatory factors.

**Figure 7:**
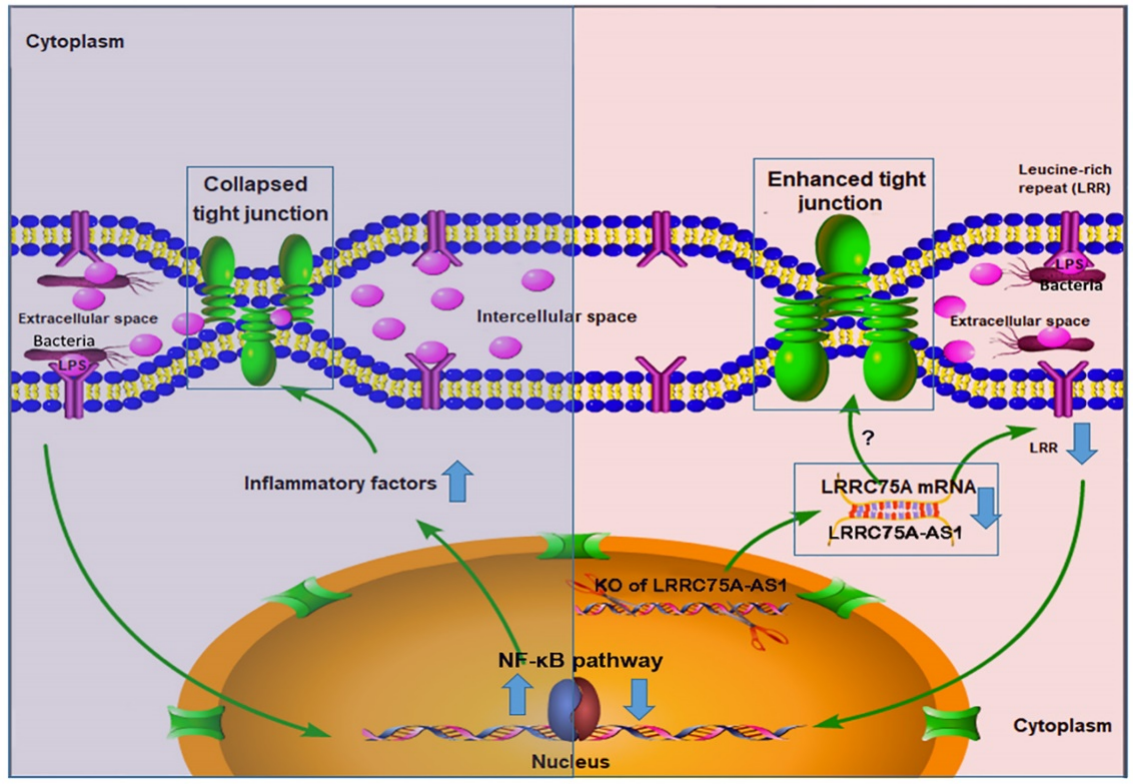
The hypothesis of how LRRC75A-AS1 functions in immune response is proposed on the base of our findings. Bacteria component, such as LPS, triggers the activation of NF-κB pathway, introducing large secretion of inflammatory factors, which collapsed the TJ structure resulting in more exposes of cell to bacteria and triggering more intensified inflammation (left). Simultaneously, LRRC75A-AS1 is downregulated, then LRRC75A was degraded because of losing the protection of base pairing with LRRC75A-AS1, leading to enhanced TJ and less antigen receptor on the surface of cell, and finally attenuating the activation of inflammation (right). Left part and right part of the mechanism contribute together to an appropriate and sound immune response of bovine mammary epithelial cells to the bacteria stimuli.

To dig deeper mechanism of how LRRC75A-AS1 works, we investigated its target gene LRRC75A, a novel and uncharacterized gene. After LRRC75A-AS1 was knocked out, LRRC75A was down-regulated. The siRNA-mediated LRRC75A knockdown suppressed the activation of inflammation, which is consistent with the result when LRRC75A-AS1was knocked out; while overexpression of LRRC75A enabled the opposite effect. Therefore, we reasoned that LRRC75A-AS1 can stabilize the LRRC75A mRNA by binding it 31, 32, 44, and when we lower the expression of LRRC75A-AS1, LRR75A mRNA was degraded more, which affected the activation of NF-κB pathway. LRRC75A has a conserved domain Leucine-rich repeats (LRRs) (cd00116), consist of 20-29 residue sequence motifs present in many proteins that is responsible for protein-protein interactions and have different functions and cellular location 45, 46. For example, toll-like receptor ectodomains possess successive copies of a LRRs motif and they may act as the structural basis of interaction between pathogenic substances and cell surface 47, 48. LRRs-containing protein has been considered as a novel pattern recognition receptor to recognize pathogen-associated molecular patterns triggering downstream immune response 49, 50. Thus LRRC75A may act as receptor on membrane, and overexpression of LRRC75A increases the LRR motif on the cell surface promoting more interaction between bacteria components, such as LPS, and its receptor constructed by LRR domain, triggering more intensified inflammatory response including the activation of NF-κB pathway and more followed secretion of various inflammatory factors, which may explain the result that less bacteria adhere to LRRC75A-AS1^-/-^ cell than control, and the result that down-regulated LRRC75A led to the less activation of NF-κB pathway while overexpressed LRRC75A contributed to the opposite. In addition, high level of inflammatory factors and bacterial toxins can disrupt TJ structure 30, 51-54. Based on these previous findings, a theory is proposed that during inflammation, bacterial antigen activates NF-κB pathway, then together with bacterial toxins, a large amount of introduced inflammatory factors results in broken TJ structure. Collapsed TJ increases cell monolayer permeability, making cells expose more to bacteria and triggering more inflammatory reaction, which facilitates the clearance of bacteria and their toxic substances but at risk of creating a positive feedback loop of overreaction. Meanwhile, LRRC75A-AS1 can control the stimulation input by affecting TJ structure through LRRC75A, restricting the development of immune response, which may be part of self-protection mechanism in bovine mammary epithelial cell (Fig. 7).

During the development of mastitis, integrity of the blood-milk barrier of alveolar epithelium maintained by epithelial TJ is broken 55, the reason of which is that inflammation changes the composition and expression of the TJ 40. In this study, we report that LRRC75A-AS1 functions as a valve that regulates the intensity of immune response by affecting TJ through LRRC75A. Decreased expression of LRRC75A-AS1 may allow the cells to response moderately upon exposure to various inflammatory stimuli, avoiding inflammatory overreaction of the cells and keeping appropriate monolayer permeability for blood-borne immune factors to enter the alveolar lumen at the same time, which suggests that LRRC75A-AS1 may serve as a potential therapeutic target in bovine mastitis treatment.

## Supplementary Material

Supplementary figures and tables.

## Authors' contributions

The study was designed by Xixi Wang and Ming-Qing Gao. The experiments were performed by Xixi Wang, Hao Wang, Ruiqi Zhang and Dan Li. Data were analyzed by Hao Wang and Xixi Wang. Ming-Qing Gao provided all the reagents and materials. Xixi Wang drafted the paper, Hao Wang and Ming-Qing Gao revised the manuscript.

## Figures and Tables

**Table 1:** Permeability rate of bovine mammary gland epithelial barrier model

Cell type	KO-Control	KO- LRRC75A-AS1
Permeability rate (%)	0.31±0.013	0.25±0.003**

Note: The permeability rate was calculated as the ratio of HRP in the 6-well plate to the total HRP initially added into the transwell chamber. **p<0.01 vs KO-Control.

**Table 2:** Adhesion and invasion percentages of *Staphylococcus aureus* to cells

Cell type	Added bacteria numbers	Adhesion (%)	Invasion (%)
KO-Control	1.0×10^7^	7.44±0.81	2.65±0.15
KO-LRRC75A-AS1	1.0×10^7^	5.68±0.31**	2.14±0.11**

Note: The adhesion or invasion percentage was calculated as the ratio of fluorescence of bacteria adhering to cells or invading into cells to fluorescence of bacteria initially added onto the cell monolayer. **p<0.01 vs KO-Control.

**Table 3:** Permeability rate of bovine mammary gland epithelial barrier model

Cell types	CD513B-1	Over-LRRC75A	Si-NC	Si-LRRC75A
Permeability rate (%)	0.25±0.0002	0.31±0.004**	0.25±0.004	0.22±0.003**

Note: The permeability rate was calculated as the ratio of HRP in the 6-well plate to the total HRP initially added into the transwell chamber. **p<0.01 vs Control (CD513B-1 or Si-NC).

**Table 4:** Adhesion and invasion percentages of *Staphylococcus aureus* to cells

Cell types	Added bacteria numbers	Adhesion (%)	Invasion (%)
CD513B-1	1.0×10^7^	7.40±0.11	2.49±0.04
Over-LRRC75A	1.0×10^7^	9.03±0.27**	2.91±0.06**
Si-NC	1.0×10^7^	4.07±0.03	2.37±0.06
Si-LRRC75A	1.0×10^7^	3.88±0.04**	1.87±0.06**

Note: The adhesion or invasion percentage was calculated as the ratio of fluorescence of bacteria adhering to cells or invading into cells to fluorescence of bacteria initially added onto the cell monolayer. **p<0.01 vs Control (CD513B-1 or Si-NC).
