# Supplementary material for: LRRC75A antisense lncRNA1 knockout attenuates inflammatory responses of bovine mammary epithelial cells
Source: Int J Biol Sci. 2020 Jan 1;16(2):251–63. doi: 10.7150/ijbs.38214 (PMC6949150; doi:10.7150/ijbs.38214)
Supplement: Supplementary file 1 — Supplementary figures and tables. [file ijbsv16p0251s1.pdf]

Table S1 A list of primers for PCR

genes	Forward primer	Reverse primer
GAPDH	AGCCGTAACTTCTGTGCTGT	ATGGCGACGATGTCCACTTT
IL-1α	CAGTTGCCCATCCAAAGTTGTT	TGCCATGTGCACCAATTTTT
IL-1β	GAGCCTGTCATCTTCGAAACG	GCACGGGTGCGTCACA
IL-6	CCACCCCAGGCAGACTACTTC	CCATGCGCTTAATGAGAGCTT
IL-8	CGATGCCAATGCATAAAAAC	CTTTTCCTTGGGGTTTAGGC
LRRC75A-AS1	CCAGCCCAAGACAAGGTAAG	AAAGGTGCCAAATCCGTTTCTT
ZO-1	GCGAAATGAGAAACAAGCACC	ATGAGTTGAGTTGGGCAGGAC
Occludin	CAGCAGCAGTGGTAACTTGGA	CCGGTCGTGTAGTCTGTTTCAT
Claudin-1	AAGACGACGAGGCACAGAAGA	GAAGGTGCTGGCTTGGGATAG
LRRC75A (344 bp ~ 500 bp)	TCACGCACGACCTCATCATC	TTCCTCTTCACCAGCCCAC
LRRC75A (1194 bp ~ 1339 bp)	TAACTCCTCTTCGGTCCTT	GGTCTCCAGATTCACAGTAG
LRRC75A for cloning	ATGGGCACCCGGCAGAC	CCTGTATTCCCCTGTCACGTCT
U6	CGCTTCGGCAGCACATATAC	ACGCTTCACGAATTTGCGTGTC
β-actin	ATCACCATCGGCAATGAGCGGTTC	CGGATGTCGACGTCACACTTCATGA

Table S2 The single-strand DNA used in the study

	Sequence of sgRNA(5'-3')	Complementary sequence of sgRNA(5'-3')
sgRNA1	CACCGTGCCGGCTCTAAAACTCTGC	AAACGCAGAGTTTTAGAGCCGGCAC
sgRNA2	CACCGAAGTTTACTCACCTCGGTCC	AAACGGACCGAGGTGAGTAAACTTC
sgRNA3	CACCGATCCGGCTTCCCATGGGGGT	AAACACCCCCATGGGAAGCCGGATC
sgRNA4	CACCGGCACCTTTTTTCTAGGATAG	AAACCTATCCTAGAAAAAAGGTGC

Note: Every sgRNA annealed with its complementary sequence and formed a double strand DNA. Red letters indicate cohesive ends for ligation. SgRNA1 and sgRNA2 were ligated into one KO plasmid, while sgRNA3 and sgRNA4 were ligated into the other KO plasmid in the study.
